# Efficient quantification of Parkinson’s disease severity using augmented time-series data

**DOI:** 10.1371/journal.pone.0319826

**Published:** 2025-04-02

**Authors:** Hua Huo, Shupei Jiao, Dongfang Li, Lan Ma, Ningya Xu

**Affiliations:** 1 Henan University of Science and Technology, LuoYang, China; Politecnico di Torino, ITALY

## Abstract

The diagnosis of Parkinson’s disease relies heavily on the subjective assessment of physicians, which depends on their individual experience and training, potentially leading to inconsistent diagnostic results. Therefore, developing an objective and efficient diagnostic method is essential to improve the accuracy and timeliness of Parkinson’s disease diagnosis. In this study, we utilized the PhysioNet dataset, a time-series dataset comprising data from 93 Parkinson’s patients and 73 healthy individuals. The dataset contains vertical ground reaction forces recorded from 16 sensors (8 per foot) during a 2-minute test at a sampling rate of 100 Hz. To address challenges such as limited dataset size, high labeling noise, and high intra-class variability, we performed data preprocessing and applied various data augmentation techniques, including jittering, scaling, rotation, permutation, magnitude warping, time warping, cropping, and linear residuals. These methods were evaluated using one-dimensional-convolutional neural network (1D-ConvNet) and one-dimensional Transformer networks. By conducting 10-fold cross-validation, we observed significant improvements in classification performance. The best data augmentation strategy achieved 90.8% accuracy, 92.0% precision, 91.0% recall, and a 91.0% F1 score in assessing disease severity. These results highlight the importance of selecting appropriate data augmentation techniques for time-series data to improve model generalization and diagnostic reliability, while also offering new insights for researchers working with sensor device data. Our results demonstrate that data-enhanced methods can significantly boost the performance of machine-learning models in the field of Parkinson’s disease diagnosis.

## 1 Introduction

Wearables are smart devices worn on the human body that utilize sensors and communication technologies to monitor and collect data on the user’s physiology, movement, environment, and other related aspects. Parkinson’s disease (PD) is one of the most common neurodegenerative diseases, typically diagnosed through clinical evaluation and neuroimaging techniques [1]. However, traditional diagnostic methods often suffer from subjectivity and delays, making early diagnosis challenging. Wearable sensors [2] such as accelerometers, gyroscopes, pressure sensors, magnetometers, and electrophysiological sensors can capture large amounts of motion data and continuously monitor the patient’s condition without real-time intervention from doctors. These devices can detect subtle changes in movement without the patient’s direct awareness, [3,4] providing strong support for the early diagnosis of PD. With the advancement of science and technology, the development of high-sensitivity wearable devices has further promoted research into PD [5]. Traditional PD diagnosis relies on subjective assessment, whereas the combination of deep learning (DL) and wearable sensors offers new avenues for objective and accurate diagnostic and monitoring tools [3]. Digital biomarkers have shown significant potential in assessing disease severity and monitoring treatment outcomes, with sensor technologies greatly improving the precision of disease monitoring [6]. Wearable devices have enhanced the timeliness and accuracy of monitoring, while digital technologies show great promise in disease tracking, personalized treatment, and remote management [7]. Additionally, digital biomarkers hold broad prospects in quantifying symptom severity and evaluating treatment effectiveness [8]. Since PD is a progressive condition, its symptoms are often subtle in the early stages. Automated severity estimation can help detect changes in the condition early, especially when symptoms worsen. Inertial data, such as accelerometer and gyroscope readings, is typically used to measure overall movement changes, but it cannot provide detailed information about the specific contact between the foot and the ground. Therefore, pressure data from the feet offers more direct and detailed feedback [9] in gait analysis.

However, despite the progress brought by digital biomarkers and wearable technologies, challenges remain, such as sample and methodological heterogeneity and the lack of public datasets [10]. Current major challenges include insufficient data standardization, small sample sizes, algorithm interpretability, and device consistency [3]. Another challenge is to design fast and accurate modeling algorithms to fine-tune the analysis of data generated by wearable devices, especially when dealing with the correlation between different sensors and timing considerations. Therefore, our research focuses on how to utilize the limited resources of plantar pressure time-series data acquired by the sensor for Data Augmentation (DA) to obtain more usable data for gait analysis, especially in the context of grading the severity of PD. Augmentation methods for image data [11] (e.g., brightness adjustment, panning, flipping, etc.) have been widely used in computer vision, and these methods improve the training effect and generalization ability of the model by increasing the diversity and quantity of data. However, similar DA methods have been applied less in sensor-acquired time series data, even though these methods could theoretically improve the performance of time series models by introducing diversity as well. This imbalance may be due to the unique continuity and dependency of time series data, making traditional image augmentation methods difficult to apply directly. Consequently, it is important to explore and develop augmentation methods applicable to time series data, especially in areas that require high accuracy and robustness, such as health monitoring and disease diagnosis.

We aim to explore DA methods for footwear time series data to provide more reliable input for the model. Another challenge is creating simplified, effective network models. Current models are complex, and we seek ways to reduce their complexity while maintaining accuracy. Simplifying the model reduces computation and training time, improving efficiency in real-world applications. By optimizing the model structure and reducing parameters, we hope to maintain good performance with plantar pressure data, which is important for early Parkinson’s diagnosis.CNNs [12] capture local features but struggle with time series data. In contrast, Transformers [13] handle long-range dependencies well and perform better with long sequences. The multi-head attention mechanism enhances efficiency, allowing the model to handle larger datasets. Therefore, we chose Deep CNN and Transformer models to process plantar pressure data and validated the DA method through experiments.

Our contributions in this paper are mainly as follows:

- **DA:** For the time series data acquired by wearable devices, we used multiple DA methods based on a limited dataset to verify its validity in a single or combined form.
- **Feature Selection:** We performed feature selection for plantar data sensors to facilitate later feature selection, thereby reducing unnecessary data information and minimizing model redundancy.
- **Excellent Performance:** By introducing a new DA method to process the plantar data of Parkinson’s patients and normal people, we achieve efficient PD severity grading. Experiments have demonstrated that our proposed method performs excellently in terms of performance and achieves significant results.
- **Simplified Model:** We verified that a single model can also achieve optimal results through DA techniques. This finding not only reduces the complexity of the model, but also simplifies the overall architecture, improving operational efficiency and practicality.

The structure of our paper is as follows: Sect 2 reviews the latest advancements and related work in DL for PD research. Sect 3 introduces the DA methods and experimental models used in our study. Sect 4 presents and analyzes the experimental results. Finally, Sect 5 summarizes the strengths and weaknesses of our work and outlines future research directions.

## 2 Related works

### 2.1 Wearable sensors in health monitoring

In recent years, the impact of load measurement, training adaptation, and injury prevention strategies in sports has been the subject of extensive research, particularly the impact of monitoring and analyzing training loads in athletes on optimizing performance, reducing injury risk, and aiding in the recovery process [14,15]. Many studies have focused on using wearable devices for real-time monitoring and assessment of gait and tremor in PD patients. For example, study [16] developed a smartphone-based measurement method to assess the severity of PD, symptom fluctuations, and the response to dopaminergic treatment. Using a smartphone application, patients remotely completed five tasks: voice, finger tapping, gait, balance, and reaction time. Bamberg et al. [17] developed an long-lasting wearable system “Gaitshoe” and used it to provide quantitative gait analysis beyond the scope of a traditional sports laboratory. GaitShoe was analyzed and validated to have a strong ability to detect heel strike and toe-off, as well as estimate foot orientation and position. In [18], wearable devices (such as accelerometers, gyroscopes, and other sensors) were used to collect patients’ motion data, particularly gait and tremor symptoms. DL methods, such as CNN and Recurrent Neural Networks (RNN), were applied to analyze the collected sensor data. The DL models are capable of extracting important features from the raw data, and subsequently predicting the severity of the patient’s condition. By using DL models to process motion data in real-time, this approach enables the monitoring and assessment of PD severity in patients. The study [19] used 16 biomedical speech features as the input dataset, with the total UPDRS (Unified Parkinson’s Disease Rating Scale is a tool used to assess the severity of PD symptoms. It helps doctors evaluate the patient’s motor skills, daily functions, and other symptoms to monitor the disease’s progression) score as the output variable. The classification accuracy for the training and test sets were 94.44% and 62.73%, respectively.

Although these methods show potential in assessing the severity of PD, they face various challenges, including reliance on data quality, individual differences, and computational resources. Therefore, expanding the dataset has become a key factor in improving the effectiveness of these methods.

### 2.2 Foot pressure analysis in Parkinson’s disease

The feasibility and applicability of objective gait measurements based on wearable sensors in PD have been demonstrated to reach a relatively mature processing technique in both large-scale clinical studies and individual patient care. Schlachetzki et al. [20] analyzed a cross-sectional study of 190 patients with PD and 101 age-matched patients and revealed significant spatiotemporal gait parameter differences and corresponding their results to physician scores successfully demonstrated the clinical applicability of wearable sensor-based gait analysis, providing high biomechanical resolution for gait disorders in PD.

With the development of machine learning (ML), researchers have also designed a variety of different models to process Parkinson’s plantar time series data, which are designed to improve the accuracy of detection and prediction of the disease, thus helping patients to get better treatment and management. Among these models, CNN, Transformer, and LSTM have been widely used and studied due to their powerful ability to process time series data and capture complex patterns. Imanne El Maachi et al. [21] used a 1D-ConvNet to construct a DNN classifier. The model processes 18 one-dimensional signals of vertical ground reaction forces (VGRF) measured from foot sensors. Their algorithm validated the model on the UPDRS grading scale, which is the first model to utilize the UPDRS scale to classify the severity of PD. Due to the need for large datasets in DL algorithms, the study divides each walking session into segments of 100 time steps, with a 50% overlap between segments. Each segment is labeled with the corresponding class for training. The segmentation process is carried out within each fold, and segments from the same subject are not shared between the training and validation sets. The final classification accuracy for disease severity is 85.3%, with a total of 64,468 segments in the dataset. Duc Minh Dimitri Nguyen et al. [22] devised a Transformer-based spatial-temporal network structure to process Parkinson’s plantar gait data, and proved them to be effective in extracting relevant features from one-dimensional signals extracting relevant features is effective. Structurally they decoupled temporal and spatial information to minimize the complexity of the model. Their architecture uses a temporal converter, a dimensionality reduction layer to reduce the dimensionality of the data, as well as a spatial converter, two fully connected layers, and an output layer for final prediction.

Moreover, the study [23] introduces a spatio-temporal capsule network to classify Parkinson’s disease severity using gait data. The experiment used a Ga dataset with dimensions 19×100, where 19 represents features and 100 represents time steps. The model achieved 100% accuracy for healthy individuals and 82% for patients with severity level 3. The model proposed by Aşuroğlu, Tunç, et al. [24] combines CNN and locally weighted random forests, using GRF sensors with a 100 Hz sampling rate. Data preprocessing removed the initial and final portions of the data and outliers were eliminated using a median filter, improving the accuracy of gait feature representation. The final model achieved a correlation coefficient of 0.897, root mean square error (RMSE) of 3.009, and root mean square (RMS) of 4.556. Shcherbak et al. [25] proposed a method based on wearable sensors and ML to distinguish between healthy controls and patients with PD stages 1 and 2. The study designed 11 common movements and collected data using commercial accelerometer, gyroscope, and magnetometer sensors from 113 participants. By applying ML techniques (feature extraction, dimensionality reduction, and classification) to analyze the data, the accuracy of early PD diagnosis was significantly improved. The best results showed F1 scores of 0.78 and 0.88 for PD stages 1 and 2, respectively. This study [26] combines ML methods to classify PD and essential tremor (ET) based on balance and gait features collected through wearable sensors. The ML model achieved an F1 score of 0.48 for PD classification, demonstrating the practicality of this approach in distinguishing different motor disorders. In study [27], 74 patients visited the laboratory every three months, completing a total of seven visits. Data on walking (2 minutes) and postural sway (30 seconds, eyes closed) were collected using six inertial measurement unit sensors. Based on this data, the MDS-UPDRS-III scale was estimated. The performance of each model was evaluated using the average RMSE on the validation set through five-fold cross-validation analysis. The results showed that the best-performing model achieved an average RMSE of 10.02. However, due to the small size of the Ga dataset, we believe the results of this study may be influenced by the limited data volume, thus lacking broader applicability to real-world scenarios.

Although existing studies have made progress in assessing PD severity using gait and motion data, challenges such as limited sample size, feature extraction constraints, model performance limitations, and individual variability remain. To address these issues, we experimented with eight DA methods to capture complex spatiotemporal features and enhance model generalization. Our study not only overcomes the limitations posed by small datasets but also provides new insights into time-series DA, laying a solid foundation for the practical application of PD severity assessment.

### 2.3 Time series data angmentation techniques

DA is an essential preprocessing step for achieving optimal performance in DL methods [12,28]. In image processing, common techniques include rotation, flipping, cropping, scaling, and adding noise [29]. In speech recognition, frequently used methods involve time warping, frequency masking, adding background noise, and pitch shifting [30]. These approaches effectively increase data diversity, enhancing the model’s generalization and robustness. However, the application of DA methods for time series is still relatively limited, primarily focusing on basic techniques such as time warping, jittering, and cropping. While these methods improve model performance to some extent, there remains significant room for development compared to image [11] and speech [19] processing. Therefore, exploring richer and more effective augmentation techniques for time series data is crucial for improving model performance. In order to better utilize sensor data, there is an urgent need to develop DA techniques that can handle complex temporal patterns and multivariate features to improve the generalization ability and robustness of models.

With the increasing application of generative adversarial networks in the field of image generation, some researchers have also focused the method on time series data. For example, Li et al. [31] proposed a Transformer-Based Time-Series Generative Adversarial Network (TTS-GAN), a generative model, a Transformer-based Generative Adversarial Network (GAN) model for synthesizing virtual data with the same length as the original time series. Subsequently, Yoon et al. [32] developed TimeGAN, a generative method for multivariate time series data. Through experimental validation, TimeGAN shows better results in maintaining data diversity and accuracy compared to other GAN. However, despite the advantages demonstrated by GAN networks in certain time series tasks, the core challenge in generating data is to ensure that the generator and discriminator reach a Nash equilibrium [33]. This equilibrium is sometimes difficult to achieve consistently in the presence of insufficient data, leading to inconsistent quality of data generated by the model. In addition, due to the complexity of the GAN training process, its stability is often difficult to ensure, making the generated virtual data may deviate from the desired results.

Window cropping or slicing is a time series DA technique. [34] proposed a method to generate new samples by extracting continuous subsequences from the original series. [35] compared it to cropping in computer vision, where adjustable-length slices are randomly extracted from the original series for classification. The labels of the slices are the same as those of the original series, and majority voting is used for classification during testing. A perturbation and ensemble-based method is proposed in [36], which generates new time series using Dynamic Time Warping (DTW) and performs ensemble via a weighted version of Barycentric Averaging (DBA) algorithm. This method improves classification performance on some UCR datasets. Alle et al. [37] designed LPGNet, which pre-processes time-series data with down-sampling, normalization, and linear predictive analysis, after which linear predictive residuals (LPRs) are used to extract discriminative patterns from gait recordings, and a 1D-ConvNet and depth-separable CNN is used to diagnose class recognition. However, these processes may lead to the loss of valuable information, especially when the data is noisy or the sampling frequency is low, and the performance of the model may be degraded. Terry T. Um et al. [38] studied wearable sensor data and proposed DA methods based on signal transformation. They used these augmentation methods in conjunction with CNN models to classify the motor states of PD patients. Experimental results show that appropriate DA improves the classification performance from 77.54% to 86.88%, validating the effectiveness of the DA methods. In study [39], which evaluated the impact of using HOAs (Hand Optimization Algorithms) to expand the training dataset and transferring weights from unsupervised pretraining on classification performance. The results showed that after expanding the dataset and pretraining on hand movement tasks, the model’s accuracy improved by 12.2%. This indicates that DA and pretraining effectively enhanced the model’s classification performance.

These methods have achieved certain successes in time series DA, but there are still some limitations. Techniques like DTW and DBA perform well on some datasets, but their applicability is narrow and cannot be widely used for complex or multivariate time series data. These methods are computationally expensive, require long training times, and may not be suitable for real-time or large-scale data applications. Meanwhile, many augmentation techniques have limited capabilities in modeling spatiotemporal features, failing to effectively capture the complex dynamic changes in the data. Finally, some methods are optimized for specific models and lack universality, resulting in significant performance variations across different models. Therefore, we aim to explore more flexible and diverse augmentation methods to better adapt to complex data structures and noisy environments, enhancing the model’s generalization ability and stability.

## 3 Methods

**Fig 1:**
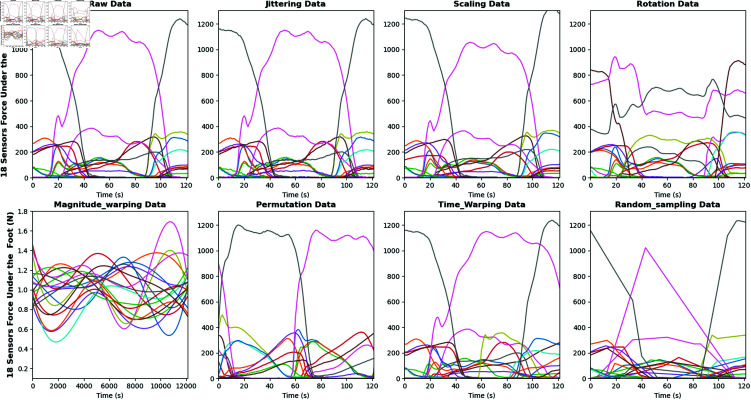
This figure shows seven DA methods we have adopted, namely Jittering, Scaling, Rotation, Magnitude_warping, Permutation, Time_warping, and Random_sampling, note that these methods can be freely combined.

In our study, we focused on how to accurately assess the severity of PD in patients based on plantar sensor data. This study utilized a publicly available dataset that recorded VGRF data from subjects walking on a flat surface at their natural gait for approximately 2 minutes. These data were obtained from 16 sensors on the soles of the feet, and the output signals from each sensor were digitized and recorded at a sampling rate of 100 times per second. To fully characterize gait, two composite signals were recorded, each representing the sum of outputs from eight sensors on each foot. This approach ensures precise motion capture while providing a rich dataset for analysis. Using various DA methods, we significantly enhanced the model’s performance on a limited time series dataset. Our DA process is illustrated in Fig 1.The methods included Jittering, Scaling, Rotation, Permutation, Mag_Warping, Time_Warping, and Cropping. Additionally, we examined the impact of linear prediction residuals (LPR) at different scales. By comparing residuals across scales, we better evaluated their influence on model training and predictions. This study not only highlights the role of residuals in DA but also refines the analytical framework, ensuring the model’s robustness and accuracy. The code can be found in here.

### 3.1 DA methods

This paper introduces eight DA methods for wearable sensor data, laying a foundation for future research. These methods are based on seven independent techniques that can be flexibly combined to achieve DA. As shown in Fig 1, we employ seven independent methods for time series DA, which can be used in any combination. Below is a brief description of our specific implementation of DA:

- *Jittering:* Using a normal distribution to generate random noise, with a mean of 0 and a standard deviation controlled by the parameter sigma, the noise is added to the original data to produce the augmented data. Then, the function is applied to the data for augmentation, with sigma set to 2 to control the noise intensity.
- *Scaling:* By generating a random scaling factor that follows a normal distribution and applying it to the original data, each row of data is multiplied by this scaling factor. The mean of the scaling factor is 1, and the standard deviation is controlled by sigma, which defaults to 0.1, thus controlling the degree of variation when augmenting the data.
- *Rotation:* The average value of each feature group is calculated as the rotation axis vector, which is then normalized to a unit vector. A random rotation angle is generated, and based on this axis vector and angle, a rotation matrix is created. The rotation matrix is applied to each feature group to obtain the rotated data.
- *Magnitude Warping:* The function generates random control points in the feature dimension of the data (based on a normal distribution), and then uses these control points to generate random curves. These curves apply deformation to the feature data through interpolation. The parameter sigma controls the magnitude of the curve’s variation, while knot determines the number of control points for the curve.
- *Permutation:* The function first randomly divides the input data into multiple segments, with the number of segments determined by the nPerm parameter, and the minimum length of each segment controlled by the minSegLength parameter to ensure each segment is sufficiently long. Then, these segments are randomly shuﬄed, and the rearranged data is returned.
- *Time Warping:* The time steps of the data are distorted by generating random curves and applying cubic spline interpolation. First, random control points are generated for each feature, and cubic spline interpolation is used to construct curves based on these control points, with the variation of the control points controlled by the sigma parameter. Then, the cumulative sum of these curves is used to convert them into cumulative time steps, simulating different time deformations.
- *Random Sampling:* The function generates random time indices for each column of the data and ensures that the starting and ending time steps are valid. Then, the function uses these randomly generated time steps to resample the data, applying linear interpolation to realign the data to the new time steps.

Among these, each DA method has its unique advantages. Jittering, by adding random noise, improves the model’s generalization ability and reduces the risk of overfitting. Scaling helps the model adapt to data changes of different magnitudes, enhancing its ability to handle various data scales. Rotation increases data diversity by altering the time order of the data, enabling the model to better cope with sequence changes in time series. Magnitude warping enhances the model’s robustness by nonlinearly adjusting the data’s magnitude, allowing it to adapt to fluctuations of different amplitudes. Permutation increases data diversity, helping the model handle changes in the order of the data. Time warping improves the model’s ability to adapt to time variations by simulating changes in time scales. Random sampling enhances data diversity by selecting different segments from the time series, helping the model recognize and process different parts of the data.

**Fig 2:**
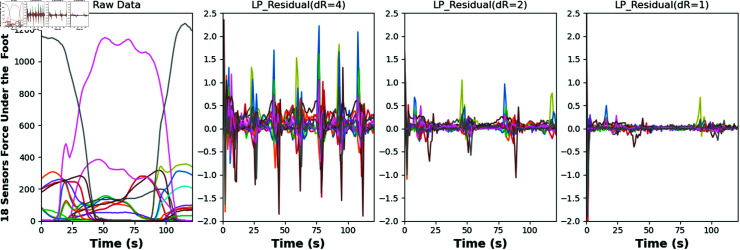
Based on our time series data(first from the left), the visualizations of linear residual predictions under different decimation rates (next three) show that as the decimation rate decreases, the range of residual variations also becomes smaller.

The use of these methods can significantly enhance the effectiveness of model training, enabling it to handle more complex and variable time series data, and improving the generalization ability and robustness of the model. In addition, the DA method we have taken is LPR. This is a concept in signal processing and time series analysis where the signal is predicted by using a linear model and then analyzing the difference between the original signal and the predicted value. This method helps in capturing and understanding the fine structure in the signal. The LPR is the difference between the original signal and its estimated value using a linear prediction model. The linear prediction model estimates the future values of a signal based on its past values using the following formula:


$$\begin{array}{ccc} \hat{x}\left(n\right)=\overset{p}{\underset{k=1}{\sum }}a_{k}x\left(n-k\right) & & \end{array}$$ (1)

where $\hat{x}\left(n\right)$ is the predicted value of the signal at time *n*, *a_k_*is the linear prediction coefficient, x (n−k)is the past value of the signal, and *p* is the order of the model. The LPR is the difference between the original signal and its predicted value. The residual formula is as follows:


$$\begin{array}{ccc} e\left(n\right)=x\left(n\right)-\hat{x}\left(n\right) & & \end{array}$$ (2)

where e (n)is the linear prediction residual at time, x (n)is the value of the original signal at time. And $\hat{x}\left(n\right)$is the predicted value of the linear prediction model at time. In summary, the linear prediction residual formula can be expressed as:


$$\begin{array}{ccc} e\left(n\right)=x\left(n\right)-\overset{p}{\underset{k=1}{\sum }}a_{k}x\left(n-k\right) & & \end{array}$$ (3)

shows that the LPR is the difference between the original signal and its estimated value by a linear prediction model. By analyzing the residuals, nonlinear components and anomalies in the signal can be revealed, which is important for signal processing, time series analysis, and various application areas. As shown in Fig 2, this is the visualization of our data after LPR processing. We first loaded the original gait data file and extracted the data using an extraction factor (decimationRate) of 4, 2 and 1, respectively.

Specifically, for each dataset, we first apply mean filtering and smoothing to the data from each sensor, then perform normalization and decimation. After that, we compute the predicted values using linear prediction coefficients and generate the prediction residual matrix.

### 3.2 Model based on CNN

**Fig 3:**
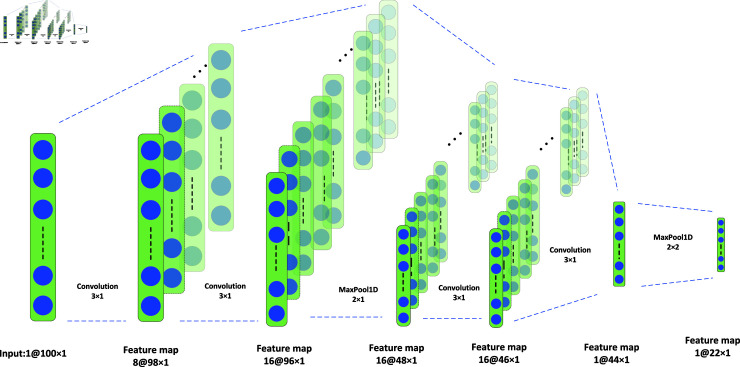
We designed a 1D-ConvNet model. This model consists of four convolutional layers and two max-pooling layers. Each channel uses this convolutional network to process the information features of different channels.

Next, we verify the effectiveness of our DA using two classical network models respectively. Firstly, the detailed structural design of our 1D-ConvNet is shown in Fig 3. This 1D-ConvNet accepts input data of shape (100, 1), and passes sequentially through two convolutional layers (with 8 and 16 filters, respectively, and kernel size of 3, using the SELU activation function, as ), one max pooling layer (with a window size of 2), and then another two convolutional layers (with 16 and 1 filters, respectively, and kernel size of 3, using the SELU activation function) and one max pooling layer (with a window size of 2).


$$\begin{array}{ccc} SELU\left(x\right)=\lambda \cdot \{\begin{array}{c} x\;\;\;\;\;\;\;\;\;\;\;if\;x>0\\ \alpha \left(e^{x}-1\right)\;\;if\;x\leq 0 \end{array} & & \end{array}$$ (4)

Subsequently, the multidimensional feature maps are flattened into one-dimensional vectors through a flattened layer, and finally, a 50-dimensional feature vector is output through a fully connected layer with 50 neurons (using the SELU activation function). The whole design aims to extract important features in the input data through convolution and pooling layers to simplify the dimensionality of the data and reduce the computational complexity, and integrate the features through a fully connected layer to generate the final feature representation. During the training process, to obtain more data, we segmented the data at the individual level, and each walk was divided into 100 time-step segments with 50% overlap, which also allowed us to adopt a majority voting mechanism through segment classification to ultimately determine the severity level of PD.

### 3.3 Model based on transformer

**Fig 4:**
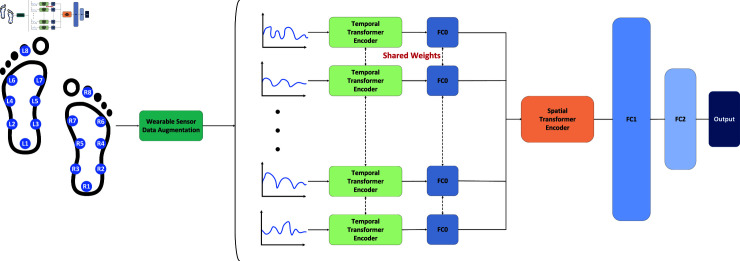
The overall architecture design of the time-spatial transformer involves the following steps. After applying various DA, the different sensor channels are fed into the time series encoder. The vectors from each channel are then fused via a fully connected layer and sent to the spatial encoder to extract higher-level features. Finally, the output is obtained through two fully connected layers (DA means Data Augmentation).

**Fig 5:**
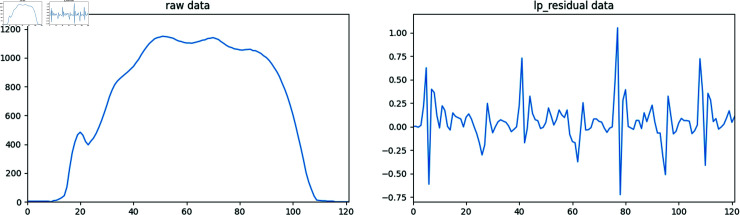
Visualization after linear residual prediction for one channel. It can be seen that the linear residual prediction compresses the range of pressure values in the time series data, reducing the range of residual fluctuations.

**Fig 6:**
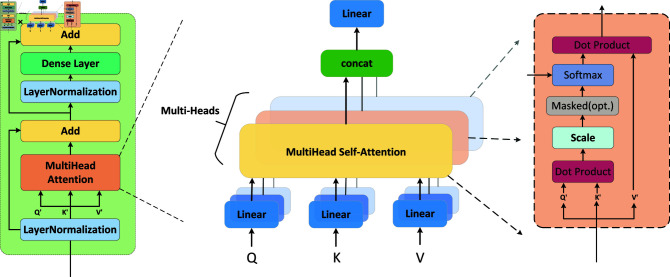
The specific implementation of the encoder’s internal structure. The structure is the same in both the time encoder and the spatial encoder. The difference lies in the fact that in the process of processing the spatial encoder, we introduce positional information to enable it to capture the interrelation between different sensors.

Model two (shown in Fig 4) begins with a variety of DAs and preprocessing of the data. Fig 5 shows the visualization results for one channel (the left foot total force channel as an example) that was processed with LPR. Next, the data was split into 18 (16 sensor channels + 2 additional channels) parallel inputs into the network. Each sensor channel is first processed through a time encoder to capture temporal dependencies. The specific design of our encoder is shown in Fig 6. First, the input data is processed through layer normalization, which helps ensure that each feature maintains consistent statistical properties during processing. Next, using the MultiHeadAttention mechanism, the encoder is able to simultaneously attend to information at different locations in the input data and perform a dropout operation after calculating the attention weights to enhance the generalization ability of the model. The attention output is then summed with the original input through residual connections, a step that helps mitigate the gradient vanishing problem and allows for a smoother transfer of information between different layers. After completing the attention mechanism, layer normalization is again performed to prepare the data for subsequent Dense layer processing. This feedforward network usually consists of fully connected layers containing activation functions for nonlinear transformation and feature extraction of the data. Eventually, the results of the feedforward processing are added to the input of the residual connection to obtain the output of the encoder. This design not only improves the performance and efficiency of the model, but also helps to capture complex relationships and features in the input data, thus laying the foundation for accurate execution of subsequent tasks.

This split-channel design maintains the independence of each channel and ensures that the data inputs obtained from different sensors are theoretically sound. After the data processing is completed, considering the importance of positional information in the plantar pressure sensors, we employ a spatial encoder design to further process and fuse the extracted feature data. This helps to capture and understand the deep information and complex patterns in the plantar pressure data, thus reflecting the dynamic changes of the plantar more accurately. Ultimately, by connecting two linear layers, we obtain the output of the multiclassification hierarchy.

It is worth mentioning that the DA processes (including data impact factors and sampling rates) in Model 1 and Model 2 are identical, ensuring that both models receive the same input data. However, in terms of preprocessing, Model 2 differs from Model 1 in that the encoder of Model 2 incorporates position encoding. By introducing position encoding, Model 2 is better able to capture the temporal features of the time series, thereby enhancing its ability to model temporal dependencies. This improvement helps the model to more accurately capture the relationships between different time steps when processing time-series data with temporal patterns. During the validation process, a specific approach was adopted to ensure the independence of subjects. When dividing the training set and the validation set, the data was distinguished into a control group and a Parkinson’s disease group. Based on the specified number of folds, a portion of subjects was selected, and then the range of corresponding segments of these subjects was determined. All the records of the selected subjects were either entirely included in the validation set or the training set, avoiding the dispersion of records from the same subject into different sets. Finally, the cumulative number of segments in the validation set and the training set was calculated. In this way, in each fold of cross - validation, all the gait records of the same subject could be ensured to be assigned to the same set, effectively avoiding result biases and poor model generalization ability caused by randomly allocating different records of the same subject to the training set and the validation set. For the data of the control group and the Parkinson’s disease group, a portion of patients was selected at a ratio of 1/10 respectively. All the data segments of these selected patients were integrally included in the validation set, while the data segments of the remaining patients were included in the training set. This division method effectively ensured that all the data records of the same subject would only appear in either the training set or the validation set, avoiding potential result biases caused by the dispersion of different records of the same subject in two sets, thus ensuring the independence of subjects.

## 4 Experiments and results

In the next sections, we will describe our experimental procedure in detail and analyze and interpret its results. First, we will introduce the dataset used and analyze the gait feature information of the dataset through visualization to show the differences between Parkinson’s patients and healthy subjects more clearly. Second, we will try multiple DA methods on different models to validate their effectiveness. Subsequently, we will explore the detailed debugging work performed during the training of the models. Finally, to reduce the complexity of the model, we try to perform feature selection based on the importance of the channels in order to simplify the model. This step helps to improve the interpretability and generalization of the model and provides a valuable reference for future model design.

### 4.1 Datasets and metrics

In this study, we used the publicly available dataset PhysioNet [40]. This dataset was contributed by several researchers including Yogev et al. [41], Hausdorff et al. [42] and Frenkel-Toledo et al. [43].The PhysioNet dataset covers data from three different gait patterns including walking on flat ground, rhythmic auditory stimulation (RAS) walking and treadmill walking. Detailed descriptions of these datasets are shown in Table 1. Divided by the number of steps walked, as in Table 2, a total of 306 gait records were collected, of which 214 (70%) were from PD patients and 92 (30%) were from healthy individuals. Table 3 shows the total number of healthy individuals and PD patients in each dataset and their corresponding severity levels, which were determined according to the H&Y (Hoehn-Yahr) scale.

**Table 1:** Statistics of participants across the three datasets.

Dataset	Group	Subjects	Male	Female	Age (years)		Height (meter)	Weight (Kg)
					**Mean ± SD**	**Range**		
Ga [41]	PD	29	20	9	61.6 ± 8.8	36–77	1.67 ± 0.07	71.3 ± 12.7
	Control	18	10	8	57.9 ± 6.7	37–70	1.68 ± 0.08	74.2 ± 12.7
Ju [42]	PD	29	16	13	66.80 ± 10.85	44–80	1.87 ± 0.15	75.1 ± 16.89
	Control	26	12	14	39.31 ± 18.51	20–74	1.83 ± 0.08	66.8 ± 11.07
Si [43]	PD	35	22	13	67.2 ± 9.1	61–84	1.66 ± 0.07	70.3 ± 8.4
	Control	29	18	11	64.5 ± 6.8	53–77	1.69 ± 0.08	71.5 ± 11.0

**Table 2:** Statistics of walking counts between Parkinson’s patients and the healthy control group.

Groups	Total walks	Normal walk		Dual task walk
		**Si**	**Ga**	**Ju**
PD walks	214	35	104	75
Control walks	92	29	25	38

**Table 3:** Statistics of the number of individuals at different disease severity levels based on the H&Y scale across the three datasets.

Dataset	Healthy	Severity2	Severity2.5	Severity 3
Ga	18	15	8	6
Ju	26	12	13	4
Si	29	29	6	0

**Fig 7:**
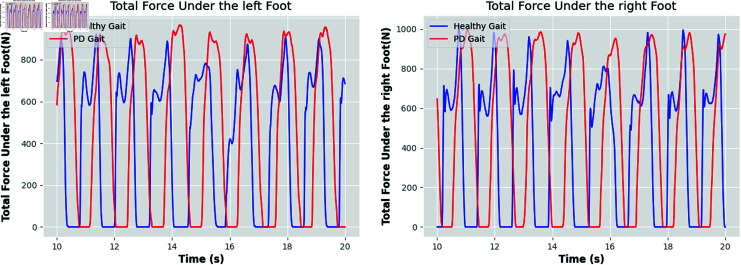
Changes in total force on the left and right feet over time for Parkinson’s patients and the healthy control group revealed that Parkinson’s patients tend to exhibit longer durations of high-pressure values. Compared to healthy participants, Parkinson’s patients spend more time on the pressure plate, which aligns with their characteristic slow gait.

**Fig 8:**
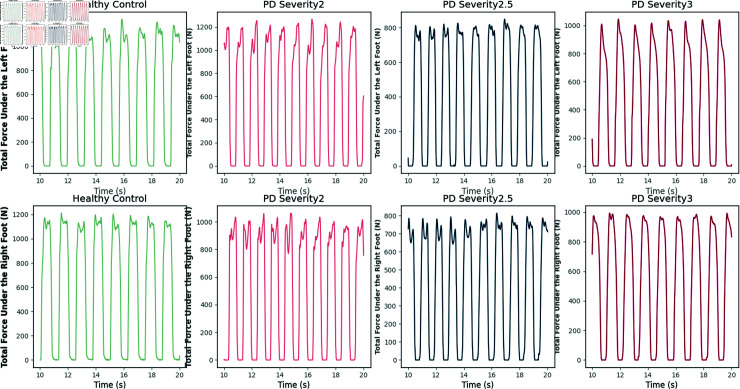
Visualization of total force on the left and right feet at different disease severity levels.

In Fig 7, we visualize the magnitude of the VGRF over time in the left and right foot for PD patients versus controls. With these visualizations, we can clearly see that the VGRF signals of PD patients and control subjects differed significantly in peak value, amplitude, shape, and timing. Specifically, the VGRF signals of the control subjects showed a more organized and consistent pattern. These signals had stable amplitudes and regular shapes with predictable timing. This consistency suggests that control subjects have good gait symmetry and stability during gait. However, the situation is different in PD patients. As the severity of PD increased, the deviations in their VGRF signals became more pronounced, as shown in Fig 8. These signals show greater variability in peak, amplitude, and timing, and tend to exhibit greater asymmetry. For example, there is a significant difference in the VGRF signal between the patient’s left and right feet, and the force exerted by the right foot may be significantly less. This asymmetry and variation not only reflects gait abnormalities, but may also result in significant impairment of the patient’s mobility in daily life.

This increase in VGRF signal deviations may be due to the lack of stability and coordination during gait in patients with PD, causing their gait patterns to become more irregular. As the disease worsens, these deviations may be further exacerbated, resulting in a greater negative impact on the patient’s daily life and functional abilities. To cope with these changes, patients with PD may require more intensive treatment and management to help them maintain a better quality of life and mobility.

We used Precision, Recall, F1, and Accuracy to evaluate our model, where the control group was identified as the negative (N) group and the Parkinson’s group was identified as the positive (P) group, and the following are the formulas for the four metrics that we used:


$$\begin{array}{ccc} Precision=\frac{TP}{TP+FP} & & \end{array}$$ (5)

$$\begin{array}{ccc} Recall=\frac{TP}{TP+FN} & & \end{array}$$ (6)

$$\begin{array}{ccc} F1=2\times \frac{Precision\times \text{Re}call}{Precision+\text{Re}call} & & \end{array}$$ (7)

$$\begin{array}{ccc} Accuracy=\frac{TP+TN}{TP+TN+FP+FN} & & \end{array}$$ (8)

Where,

- TP: The number of true positives
- TN: The number of true negatives
- FP: The number of false positives
- FN: The number of false negatives

### 4.2 Parameter analysis experiment

We conducted experiments on various parameter configurations for 1D-ConvNet, see Table 4. The results indicate that the configuration of Model 1 performs best: Conv1D settings are (8,3), (16,3), (16,3), (1,3), with SELU as the activation function, dropout set to 0.1, learning rate at 0.01, and batch size of 200. Under this setup, the model achieves Precision, Recall, and F1 scores of 0.85, 0.84, and 0.84, respectively, demonstrating excellent classification ability and generalization performance.

In comparison, Model 5, which uses larger convolution kernels, expands the range of feature extraction but shows slightly lower performance. This suggests that excessively large kernels may hinder effective capture of local features. Additionally, Model 6, which uses Tanh as the activation function, experiences a significant drop in F1 score to 0.74, further supporting SELU’s suitability for this task. The batch size analysis also confirms that a setting of 200 outperforms smaller batch sizes, leading to more stable gradient updates and improved model performance. In summary, the parameter configuration of Model 1 outperforms other combinations, highlighting its strong adaptability and advantages for the given task.

**Table 4:** Experimental analysis of 1D-ConvNet under various parameter settings.

Model	Conv1D(1)	Conv1D(2)	Conv1D(3)	Conv1D(4)	Activation	FC	Drop_rate	LR	Batchsize	Precision	Recall	F1-score
1	(8,3)	(16,3)	(16,3)	(1,3)	selu	(100,20)	0.1	0.01	200	**0.85**	**0.84**	**0.84**
2	(8,5)	(16,5)	(16,5)	(1,5)						0.83	0.79	0.81
3	(8,7)	(16,7)	(16,7)	(1,7)				0.02	100	0.79	0.78	0.79
4	(4,3)	(8,3)	(8,3)	(1,3)				0.03	150	0.82	0.81	0.82
5	(16,3)	(32,3)	(32,3)	(1,3)				0.05	150	0.81	0.80	0.79
6					tanh				50	0.77	0.73	0.74
7					relu			0.02	150	0.67	0.66	0.64
8						(200,20)				0.84	0.78	0.80
9						(100,10)		0.02	100	0.66	0.65	0.64
10							0.2		100	0.79	0.78	0.81
11							0.5	0.02	150	0.78	0.79	0.79

We also conducted experiments on various parameter configurations for temporal-spatial transformer. The experimental results (see Table 5) clearly indicate that the hyperparameter combination we selected (Num_blocks  =  1, Key_dim  =  100, Num_heads  =  2, Drop_rate  =  0.3, LR  =  0.01, Batchsize  =  100) significantly outperforms other configurations. In terms of performance metrics, this combination achieved a precision of 0.82, recall of 0.79, and F1 score of 0.79, all of which are higher than those of other setups. Compared to multi-layer Transformers (e.g., Num_blocks  =  2), the single-layer Transformer is more efficient at capturing key features, avoiding overfitting, and saving training time. The choice of feature dimension (Key_dim  =  100) and the number of attention heads (Num_heads  =  2) strikes a good balance between model complexity and data suitability. Higher feature dimensions (e.g., Key_dim  =  256) may lead to performance degradation. The selected dropout rate (Drop_rate  =  0.3) effectively reduces overfitting while maintaining the model’s learning capacity. A lower learning rate (LR  =  0.01) ensures smoother optimization, while a larger batch size (Batchsize  =  100) enhances training efficiency and model stability.

Compared to other parameter combinations, our choice simplifies the model structure and optimizes hyperparameter settings, significantly improving the model’s generalization ability and stability. This results in the best overall performance with a limited sample size, further demonstrating the importance of appropriate parameter selection for model optimization.

**Table 5:** Experimental analysis of time-spatial transformer under various parameter settings.

Model	Num_blocks	Key_dim	Num_heads	Drop_rate	LR	Batchsize	Precision	Recall	F1-score
1	1	100	2	0.3	0.01	100	**0.82**	**0.79**	**0.79**
2	2						0.80	0.77	0.78
3		64				50	0.79	0.76	0.78
4		128				50	0.81	0.80	0.79
5		256				50	0.80	0.81	0.79
6			4				0.76	0.75	0.76
7				0.5	0.02		0.79	0.77	0.78
8	2	64	4	0.5			0.82	0.81	0.79
9	2	128	4	0.5			0.78	0.79	0.77
10	2	256	4	0.5			0.79	0.75	0.76

### 4.3 Results on different models

**Results on CNN:** To evaluate the effectiveness of the DA methods, we used eight different augmentation techniques and performed a detailed comparison of the performance of these methods with the designed 1D-ConvNet model based on four core metrics, namely Precision, Recall, F1 value and Accuracy. The results of the DA experiments are shown in Table 6, which underlines the best results for each label (e.g., health status and different disease severity) on each metric.

Overall, the augmented model significantly outperforms the unaugmented data on all labels and assessment metrics, with the Jittering method performing the best with an accuracy of 89.6%. This technique adds random noise to the data to increase the perturbation, which improves the model’s generalization ability and makes it more stable when dealing with unknown data.Scaling performs very close to the same performance, achieving an accuracy of 86.3%. By scaling the data, Scaling helps the model to better adapt to changes in the data at different scales Magnitude Warping, another effective augmentation strategy, achieves an accuracy of 85.8%. This method improves the diversity by adjusting the magnitude of the data to enhance the robustness of the model. Permutation and Time Warping methods perform less well, permutation increases the diversity of the data by disrupting the temporal order of the data, but with a slightly lower accuracy. Time Warping achieves a similar effect by nonlinearly distorting the timeline, which improves the robustness of the model, but its overall performance is still not as good as that of Jittering and Scaling. Jittering and Scaling. Rotation method performs the worst, with an accuracy of 69%. Rotation, as a time-series DA method, performed poorly, possibly because it disrupted the temporal relationships in the data. Additionally, when applied to multi-axis signals, rotation might not accurately reflect the changes in the data, potentially introducing distortion and reducing the model’s performance. In addition, the table shows the results of Random Sampling and three different strengths of regularization (lp_25, lp_50, and lp_100). The regularization treatment provides limited improvement in model performance, while Random Sampling fails to significantly outperform the other augmentation methods.

In summary, there are significant differences in the effectiveness of different DA strategies in improving model performance. Among them, Jittering, Scaling and Magnitude Warping are the best performers in several indicators, especially in accuracy and F1 value. In contrast, Rotation and other methods fail to improve the model performance effectively because they change the feature pattern of the data. This suggests that the choice of different DA strategies is crucial for model performance on time series data.

**Table 6:** The performance of our designed 1D-Convnet model with different DA across various metrics (DA means data augmentation).

Metrics	Lables	Without DA	Jittering	Scaling	Rotation	Magnitude_warping	Permutation	Time_warping	Random_sampling	lp_25	lp_50	lp_100
Precision	Healthy	0.75	0.90	0.76	0.66	0.80	0.93	0.53	0.76	0.76	0.74	0.74
	Severity2	0.84	0.88	0.83	0.67	0.84	0.82	0.85	0.80	0.83	0.89	0.84
	Severity2.5	0.87	0.91	0.93	0.65	0.89	0.84	0.92	0.95	0.87	0.85	0.91
	Severity3	0.90	0.91	1.0	0.77	0.95	0.89	0.90	0.88	1.0	0.86	0.88
	Weighted Avg	0.85	0.90	0.87	0.69	0.86	0.84	0.84	0.85	0.86	0.85	0.86
Recall	Healthy	0.90	0.93	0.93	0.63	0.93	0.90	0.93	0.87	0.87	0.93	0.97
	Severity2	0.86	0.93	0.90	0.80	0.89	0.89	0.79	0.92	0.87	0.86	0.90
	Severity2.5	0.86	0.85	0.79	0.68	0.84	0.81	0.81	0.82	0.85	0.82	0.84
	Severity3	0.67	0.80	0.81	0.40	0.69	0.64	0.70	0.56	0.79	0.80	0.56
	Weighted Avg	0.84	0.90	0.86	0.69	0.86	0.84	0.80	0.84	0.85	0.85	0.85
F1	Healthy	0.82	0.92	0.84	0.64	0.86	0.92	0.67	0.81	0.81	0.82	0.84
	Severity2	0.85	0.90	0.87	0.73	0.87	0.85	0.82	0.85	0.85	0.87	0.87
	Severity2.5	0.87	0.89	0.86	0.77	0.86	0.82	0.86	0.88	0.86	0.84	0.87
	Severity3	0.77	0.85	0.89	0.53	0.80	0.74	0.79	0.68	0.88	0.83	0.68
	Weighted Avg	0.84	0.90	0.86	0.68	0.86	0.84	0.81	0.84	0.85	0.85	0.85
Accuracy	-	0.84	**0.896**	**0.863**	0.69	**0.858**	**0.842**	**0.841**	**0.842**	**0.850**	**0.850**	**0.850**

**Results on Transformer:** As shown in Table 7 are the experimental results of the encoder based model. It can be observed from the table that the baseline model without using DA methods achieves 79% accuracy in Accuracy. In contrast, most of the DA methods we used outperform the baseline model in Accuracy, Precision, Recall, and F1. Specifically:

- Jittering The model is 89.6% accurate after processing. Jittering is a technique that adds small amounts of noise to the raw data, which helps to increase the robustness of the model and makes it better able to handle noisy data in a real-world environment.
- Scaling processing resulted in 82% accuracy of the model. The scaling technique improves the generalization ability of the model by adjusting the magnitude of the data so that it is trained on different scales.
- After Permutation processing, the model has the highest accuracy of 90.83%. Permutation techniques can improve model accuracy by increasing data diversity through changing the order of the data, particularly in multi-axis signals. By modifying the sequence of the data, permutation allows the model to learn different combinations, helping it identify new patterns. In time-series data, permutation not only enhances the data diversity but also helps the model discover dependencies in different orders, thus improving generalization and accuracy.
- In the Random Sampling processing stage, the model’s accuracy was 81.5%. Random Sampling allows the model to better cope with data imbalances by randomly selecting a subset of the original data for training.

However, not all DA methods significantly improve model performance. For example:

- After Rotation and Time Warping processing, the model fails to process the data efficiently and performs poorly. Rotation increases the diversity of the data by rotating it, but in some cases may destroy the original structure of the data. Time Warping distorts the data on the time axis by making it change in the time dimension, but this change may result in the loss of the temporal characteristics of the data.
- In Magnitude Warping processing, the model performance is the same as the baseline model, and there is no significant advantage. Magnitude Warping increases the diversity of the data by changing its magnitude, but this method retains less of the data features, and therefore fails to improve the model performance significantly.

**Table 7:** The performance of our time-spatial transformer model with different DA across various metrics (DA means data augmentation).

Metrics	Lables	Without DA	Jittering	Scaling	Rotation	Magnitude_warping	Permutation	Time_warping	Random_sampling	lp_25	lp_50	lp_100
Precision	Healthy	0.58	0.84	0.82	0.48	0.78	0.79	0.56	0.58	0.71	0.65	0.67
	Severity2	0.82	0.85	0.84	0.71	0.79	0.89	0.75	0.84	0.80	0.80	0.84
	Severity2.5	0.88	0.92	0.79	0.78	0.79	1.0	0.91	0.91	0.88	0.94	0.96
	Severity3	0.90	0.95	0.84	0.88	0.92	0.86	1.0	0.92	0.94	0.94	0.85
	Weighted Avg	0.82	0.88	0.82	0.72	0.80	0.92	0.80	0.84	0.83	0.81	0.86
Recall	Healthy	0.97	0.90	0.90	0.60	0.83	1.0	0.83	0.90	0.80	0.80	0.86
	Severity2	0.81	0.90	0.87	0.81	0.82	0.96	0.88	0.84	0.85	0.83	0.88
	Severity2.5	0.71	0.88	0.80	0.75	0.85	0.85	0.69	0.80	0.86	0.82	0.84
	Severity3	0.73	0.75	0.57	0.16	0.46	0.75	0.38	0.65	0.59	0.62	0.73
	Weighted Avg	0.79	0.88	0.82	0.70	0.80	0.91	0.76	0.82	0.82	0.80	0.85
F1	Healthy	0.72	0.87	0.86	0.54	0.81	0.88	0.67	0.70	0.75	0.72	0.75
	Severity2	0.82	1.0	0.85	0.75	0.81	0.92	0.81	0.84	0.83	0.82	0.86
	Severity2.5	0.78	0.88	0.79	0.76	0.82	0.92	0.79	0.85	0.87	0.83	0.90
	Severity3	0.82	0.84	0.68	0.40	0.61	0.80	0.56	0.76	0.73	0.74	0.79
	Weighted Avg	0.79	0.88	0.82	0.69	0.79	0.91	0.76	0.82	0.82	0.80	0.85
Accuracy	-	0.790	**0.879**	**0.820**	0.700	**0.795**	**0.908**	0.765	**0.815**	**0.820**	**0.800**	**0.851**

To summarize, through different DA methods, we can significantly improve the performance of the model, but we need to pay attention to choosing the appropriate method to avoid performance degradation in some cases. This suggests that in practical applications, the selection of DA techniques should be optimized with specific tasks and data features and different models to achieve the best results.

**Results with Other Methods:** This Table 8 demonstrates the performance comparison of different models in terms of prediction accuracy. The model we designed with DA and Spatio-Temporal Transformer techniques achieves an accuracy of 90.8%, which is a significant improvement compared to other models.

Specifically, Caramia et al.’s SVM-RBF model achieved 75.6% accuracy on the H&Y scale, which is the lowest result in the table. The Artificial Neural Network (ANN) model used by Veeraragavan et al. achieved 87.1% accuracy, while El Maachi et al.’s 1D-ConvNet under the UPDRS rating system achieved 85.3%, and 85.3% F1-score. Safwen Naimi et al. achieved 87.89% accuracy by combining CNN with Transformer architecture and performed well on H&Y grading, and 88.0% F1-score.

Compared to these models, our DA + spatio-temporal Transformer model shows an extremely high accuracy of 90.8% on the H&Y grading system, and 91.0% F1-score. This suggests that our approach not only fully utilizes DA strategies (e.g., Jittering, Scaling, etc.), but also better captures temporal and spatial features in the data by introducing the spatio–temporal Transformer model. This performance enhancement illustrates the value of combining DA with advanced modeling structures and provides strong support for further exploration of similar applications.

**Fig 9:**
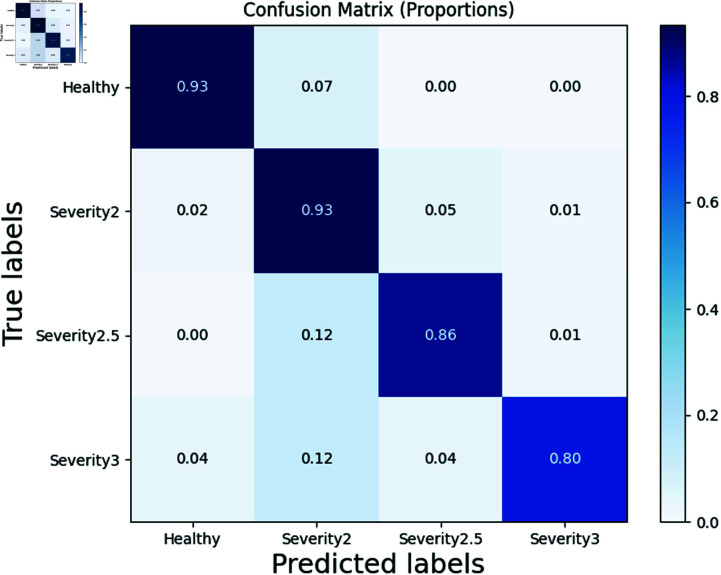
Confusion matrix based on CNN model with DA (DA means data augmentation).

**Fig 10:**
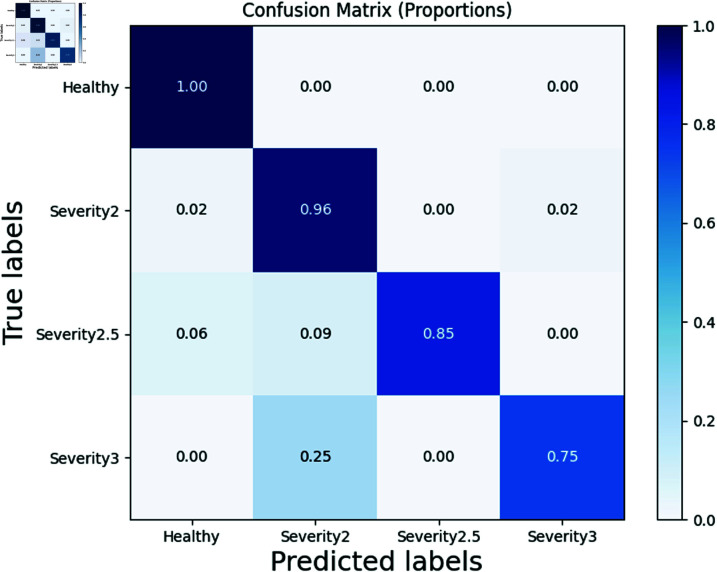
Visualization of the independent samples t-test results before and after DA (DA means data augmentation).

We present the confusion matrix results for two models in Figs 9 and 10. In a confusion matrix, the diagonal elements represent the number of samples correctly predicted by the model for each class, while off-diagonal elements represent misclassifications where one class is predicted as another. It can be observed that our models demonstrate high prediction accuracy across various classes.

Moreover, to verify the stability of the model’s performance, we conducted an independent samples t-test on the results before and after DA. The p-value we obtained from the test was 0.0326, which is less than the commonly used significance level of 0.05, indicating that there is a significant difference in the predicted values between augmented and non-augmented data. Specifically, the DA method had a significant impact on the model’s prediction results. This means that, after using DA, the model’s prediction performance significantly differs from when DA was not used. The result suggests that DA may have improved the model’s robustness and accuracy when handling different variations in the data, thereby enhancing the model’s generalization ability.

To ensure the reliability of our conclusions, we further visualized the distribution of the predicted values for augmented and non-augmented data, as shown in Fig 11. The blue bars represent non-augmented data, the red bars represent augmented data, and the purple area represents the overlap between the two. From the graph, it can be seen that, for the mild Parkinson’s prediction values (i.e., early and middle stages), the red bars (augmented data) are generally higher than the blue bars. This indicates that, after DA, our model performs better at identifying misclassifications in the non-augmented data, particularly in cases where healthy data is misclassified as disease data. DA likely improved the model’s prediction frequency and coverage, especially for low-frequency categories, further optimizing the overall performance of the model.

**Table 8:** The performance comparison of different models in terms of accuracy.

	Algorithm	Database	Preprocessing	Strategy	Stages	Accuracy (%)	F1-score (%)
Caramia et al. [44]	SVM-RBF	Hospital General Universitario Gregorio Marañon	-	-	H&Y Scale	75.6	n/a
Veeraragavan et al. [45]	ANN	Physionet	-	15-fold	H&Y Scale	87.1	n/a
El Maachi et al. [21]	1D-ConvNet	Physionet	50% overlap	10-fold	UPDRS	85.3	85.3
Safwen Naimi et al. [46]	Hybrid ConvNet-transformer	Physionet	50% overlap	10-fold	H&Y Scale	87.9	88.0
Ours (without DA)	Time-spatial transformer	Physionet	50% overlap	10-fold	H&Y Scale	79.0	79.0
Ours	DA+Time-spatial transformer	Physionet	50% overlap	10-fold	H&Y Scale	**90.8**	91.0

### 4.4 Implementation details

The parameters of each factor of the DA method we used are shown in the Table 9, and in linear residual prediction we used decimationRate extraction factors of 1, 2, and 4 for the experiments, respectively. The parameter settings of the 1D-ConvNet are shown in Fig 3.

We set the learning rate to 0.0002 during training and enabled the EarlyStopping mechanism. This callback function checks the monitored metric (val_loss) at the end of each training cycle. Training is stopped if the metric does not improve significantly (i.e., the improvement is less than min_delta) within the specified PATIENCE period. This helps prevent the model’s performance from deteriorating on the validation set and avoids overfitting. We chose the Nadam (Nesterov-accelerated Adaptive Moment Estimation) optimizer, a variant based on the Adam optimizer that combines the advantages of the Nesterov-accelerated gradient and Adam. The specific parameters are *β*1 = 0 . 9, *β*2 = 0 . 999, and *β*2 = 0 . 999. a

To ensure the independence of subjects, we guarantee that each subject’s data appears only in either the training set or the testing set, avoiding information leakage. During cross-validation, the data of each subject is appropriately assigned to different subsets, ensuring that the subjects in the training and validation sets are completely independent, thereby improving the reliability of model evaluation.

**Table 9:** Setting of parameter factors for different methods of DA in experiments (DA means data augmentation).

Factor	Jittering	Scaling	Rotation	M_warping	Permutation	T_warping	R_Sampling
sigma	2	0.1	-	-	-	0.2	-
knot	-	-	-	4	-	4	-
nPerm	-	-	-	-	4	-	-
minSeLength	-	-	-	-	100	-	-
nSample	-	-	-	-	-	-	1000

### 4.5 Feature selection experiment

We observed some interesting phenomena when we conducted ablation experiments on the data from eighteen sensors. Specifically, we compared sensors symmetrically located on the left and right feet. As can be seen from Table 10, the experimental results show that L2&R2 have the lowest accuracy, which suggests that the second sensors on both the left and right feet are the most important, likely containing critical discriminative feature information. Additionally, the accuracy of L5&R5 and L8&R8 also decreased, indicating that these sensors play a crucial role in the model, and thus these features should be given special attention during modeling. In contrast, L3&R3 have the highest accuracy, and their absence has minimal impact on overall performance, suggesting that the third sensors on both feet are of lower importance. Considering the computational resource constraints of the model, it will be worth considering removing these two features or reducing their weight in the model to optimize both computational efficiency and performance.

**Table 10:** Ablation experiments on the impact of different sensors on overall performance. The first column represents the experimental results after removing each sensor (The bold text indicates a higher level of importance).

VGRF inputs	Precision	Recall	F1	ACC (%)
L1&R1	0.870	0.858	0.856	0.858
L2&R2	0.831	0.829	0.829	**0.829**
L3&R3	0.878	0.875	0.874	0.875
L4&R4	0.870	0.863	0.860	0.863
L5&R5	0.863	0.854	0.852	**0.856**
L6&R6	0.860	0.858	0.856	0.858
L7&R7	0.866	0.863	0.861	0.863
L8&R8	0.861	0.854	0.851	**0.854**
Total VGRF(L&R)	0.865	0.823	0.861	0.863

## 5 Conclusion and future work

In this study, we experimented with various DA methods, such as permutation, jittering, scaling, and other techniques for plantar time series data, and the experimental results showed that the use of appropriate DA can significantly improve the robustness and generalization ability of the model. We chose 1D-ConvNet and spatio-temporal combined one-dimensional encoder as the experimental test models, and the results show that our data preprocessing design not only reduces the cost of computational resources, but also shortens the training time, which makes the model efficiently transferable to real-time applications. Through the experiments with the above methods, our model achieves an accuracy of 90.8% on the H&Y grading scale, and realizes a precision of 92.0%, a recall of 91.0%, and an F1 score of 91.0%. Clearly superior to existing state-of-the-art techniques, this demonstrates the validity and strength of our method. Unlike in the past, we achieve high precision while still maintaining the simplicity of the model design, which means that the model is easier to deploy and maintain in real-world applications.

In addition, we conducted a feature selection study for different sensors, aiming at effective feature screening. This study not only provides important data support for our current work, but also provides valuable inspiration for future directions. By delving deeper into these data, we will be able to identify and eliminate redundant information, enabling more accurate extraction of useful features. This will significantly enhance the model’s performance and efficiency, making it more reliable and effective in practical applications. Additionally, by optimizing the feature selection process, we will build more concise and efficient models, providing clinicians and researchers with more valuable insights and support.

Although the proposed method achieves remarkable results in feature selection and model optimization, it still has some limitations. For instance, the dataset has limited coverage, suggesting the need for broader data collection in the future. Moreover, since the dataset is primarily derived from controlled environments, the model’s generalizability is restricted. Additionally, its adaptability to dynamic changes in environments or data collection conditions requires further improvement. Compared to previous studies, this work incorporates DA techniques, which not only enhance the processing speed but also maintain overall efficiency. Unlike traditional approaches that directly use full-scale features or build complex models, this study employs a systematic feature selection process, providing valuable references and insights for future research on feature selection. In terms of computational cost, the simplified single-model approach demonstrates significant advantages: by reducing model complexity and parameters, it markedly lowers computational resource requirements and runtime while remaining resource-friendly and easy to maintain. This enables the model to operate reliably on low-performance devices, offering a more efficient and dependable solution for primary healthcare and resource-constrained settings.
